# Supplementary material for: A Novel Summer Camp Integrating Physical, Psychological, and Educational Health in Youth: The THINK Program
Source: Nutrients. 2024 Jun 12;16(12):1838. doi: 10.3390/nu16121838 (PMC11206397; doi:10.3390/nu16121838)
Supplement: Supplementary file 1 [file nutrients-16-01838-s001.zip › nutrients-3003400-supplementary.pdf]

SUPPLEMENTARY MATERIALS

The Adolescent Food Habits Checklist

1. If I am having lunch away from home, I often choose a low-fat option.

True False I never have lunch away from home

2. I usually avoid eating fried foods.
True False

3. I usually eat a dessert or pudding if there is one available.
True False

4. I make sure I eat at least one serving of fruit a day.
True False

5. I try to keep my overall fat intake down.
True False

6. If I am buying potato chips, I often choose a low-fat brand.
True False I never buy potato chips

7. I avoid eating lots of sausages and burgers.
True False I never eat sausages or burgers

8. I often buy pastries or cakes.
True False

9. I try to keep my overall sugar intake down.
True False

10. I make sure I eat at least one serving of vegetables or salad a day. True False

11. If I am having a dessert at home, I try to have something low in fat. True False I don't eat desserts

12. I rarely eat takeaway meals.
True False

13. I try to ensure I eat plenty of fruit and vegetables.
True False

14. I often eat sweet snacks between meals.
True False

15. I usually eat at least one serving of vegetables (excluding potatoes) or salad with my evening meal.
True False

16. When I am buying a soft drink, I usually choose a diet drink.
True False I never buy soft drinks

17. When I put butter or margarine on bread, I usually spread it thinly. True False I never have butter or margarine on bread

18. If I have a packed lunch, I usually include some chocolate and/or cookies. True False I never have a packed lunch

19. When I have a snack between meals, I often choose fruit.
True False I never eat snacks between meals

20. If I am having a dessert or pudding in a restaurant, I usually choose the healthiest one. True False I never have desserts in restaurants

21. I often have cream on desserts.
True False I don't eat desserts

22. I eat at least three servings of fruit most days.
True False

23. I generally try to have a healthy diet.
True False

SCORING

1 point for each '**healthy'** response. (False for questions 3, 8, 14, 18, 21. True for all others.)

Final score should be adjusted for 'not applicable' responses (questions 1, 6, 7, 11, 16, 17, 18, 19, 20, 21), and missing responses using the formula:

AFHC score = no of 'healthy' responses x (23/no of items completed).

Reference:

Johnson, F, Wardle, J & Griffith, J

The Adolescent Food Habits Checklist: reliability and validity of a measure of healthy eating behaviour in adolescents

European Journal of Clinical Nutrition (2002): ***56,*** *644-649*

The Positive Youth Development Inventory Full Version (2012)

We would like to gather some information about the things you learned while participating in this program. Your responses are completely anonymous (meaning no one will know it is you who completed this form) and voluntary (meaning you don't have to complete this form if you do not want to). You can leave any question blank, and you can also choose not to complete the questions once you begin. Nor will any answers you provide be singled out; we will look at everyone's answers together.

Please rate your agreement using: 1) Strongly Disagree; 2) Disagree; 3) Agree; 4) Strongly Agree

As a result of participating in this program:

	Strongly disagree	Disagree	Agree	Strongly agree
1. I am a good student	O	O	O	O
2. I take part in activities at my school	O	O	O	O
3. I like to learn about new things	O	O	O	O
4. I am a creative person	O	O	O	O
5. I make good decisions	O	O	O	O
6. I make friends easily	O	O	O	O
7. I feel comfortable in social situations	O	O	O	O
8. I can handle problems that come up in my life	O	O	O	O
9. I can manage my emotions	O	O	O	O
10. I can handle being disappointed	O	O	O	O
11. I am aware of other people's needs in social situations	O	O	O	O
12. I have goals for my life	O	O	O	O
13. I know what I want to do for a career	O	O	O	O
14. I am interested in learning about careers I could have	O	O	O	O
15. It is important for me to do the right thing	O	O	O	O
16. I try to do the right thing, even when I know that no one will know if I do or not.	O	O	O	O
17. I think it is important for me to be a role model for others.	O	O	O	O
18. It is important for me to do my best.	O	O	O	O
19. It is important that others can count on me.	O	O	O	O

	Strongly disagree	Disagree	Agree	Strongly agree
20. If I promise to do something I can be counted on to do it.	O	O	O	O
21. I am able to behave appropriately in most settings.	O	O	O	O
22. I am able to stand up to peer pressure when I feel something is not right to do	O	O	O	O
23. I have people in my life whom I look up to and admire	O	O	O	O
24. I have a wide circle of friends.	O	O	O	O
25. I think it is important to be involved with other people.	O	O	O	O
26. My friends care about me.	O	O	O	O
27. I feel connected to my teachers.	O	O	O	O
28. Having friends is important to me.	O	O	O	O
29. I feel connected to others in my community.	O	O	O	O
30. I have adults in my life who are interested in me.	O	O	O	O
31. I feel connected to my parents	O	O	O	O
32. When there is a need I offer assistance whenever I can.	O	O	O	O
33. It is easy for me to consider the feelings of others.	O	O	O	O
34. I care about how my decisions affect other people.	O	O	O	O
35. I try to encourage others when they are not as good at something as me.	O	O	O	O
36. Other people’s feelings matter to me.	O	O	O	O
37. I can be counted on to help if someone needs me.	O	O	O	O
38. I care about the feelings of my friends.	O	O	O	O
39. When one of my friends is hurting, I hurt too.	O	O	O	O
40. I feel good about my scholastic ability	O	O	O	O
41. I feel I am a good athlete	O	O	O	O
42. I am satisfied with how I look	O	O	O	O
43. I feel accepted by my friends	O	O	O	O
44. In general, I think I am a worthy person	O	O	O	O
45. I know how to behave well in different settings	O	O	O	O

	Strongly disagree	Disagree	Agree	Strongly agree
46. I can figure out right from wrong	O	O	O	O
47. I have close friendships	O	O	O	O
48. I can do things that make a difference	O	O	O	O
49. I take an active role in my community.	O	O	O	O
50. I am someone who gives to benefit others.	O	O	O	O
51. I like to work with others to solve problems.	O	O	O	O
52. I have things I can offer to others.	O	O	O	O
53. I believe I can make a difference in the world.	O	O	O	O
54. I care about contributing to make the world a better place for everyone.	O	O	O	O
55. It is important for me to try and make a difference in the world.	O	O	O	O

Arnold, M.E., Nott, B. D., & Meinhold, J. L. (2012). *The Positive Youth Development Inventory Full Version.*

© Oregon State University. All Rights Reserved.

Middle/High School Student Attitudes toward STEM (S-STEM) – 6-12th

There are lists of statements on the following pages. Please mark your answer sheets by marking how you feel about each statement. For example:

Example 1:	Strongly Disagree	Disagree	Neither Agree nor Disagree	Agree	Strongly Agree
I like engineering.	○	○	○	○	○

As you read the sentence, you will know whether you agree or disagree. Fill in the circle that describes how much you agree or disagree.

Even though some statements are very similar, please answer each statement. This is not timed; work fast, but carefully.

There are no "right" or "wrong" answers! The only correct responses are those that are true *for you*. Whenever possible, let the things that have happened to you help you make a choice.

Please fill in only one answer per question.

Recommended citation for this survey:

> Friday Institute for Educational Innovation (2012). *Middle/High School Student Attitudes toward STEM Survey*. Raleigh, NC: Author.

Math

	Strongly Disagree	Disagree	Neither Agree nor Disagree	Agree	Strongly Agree
27. Math has been my worst subject.	○	○	○	○	○
28. I would consider choosing a career that uses math.	○	○	○	○	○
29. Math is hard for me.	○	○	○	○	○
30. I am the type of student to do well in math.	○	○	○	○	○
31. I can handle most subjects well, but I cannot do a good job with math.	○	○	○	○	○
32. I am sure I could do advanced work in math.	○	○	○	○	○
33. I can get good grades in math.	○	○	○	○	○

34. I am good at math.	○	○	○	○	○

Science

	Strongly Disagree	Disagree	Neither Agree nor Disagree	Agree	Strongly Agree
35. I am sure of myself when I do science.	○	○	○	○	○
36. I would consider a career in science.	○	○	○	○	○
37. I expect to use science when I get out of school.	○	○	○	○	○
38. Knowing science will help me earn a living.	○	○	○	○	○
39. I will need science for my future work.	○	○	○	○	○
40. I know I can do well in science.	○	○	○	○	○
41. Science will be important to me in my life's work.	○	○	○	○	○
42. I can handle most subjects well, but I cannot do a good job with science.	○	○	○	○	○

	Strongly Disagree	Disagree	Neither Agree nor Disagree	Agree	Strongly Agree

43. I am sure I could do advanced work in science.	○	○	○	○	○

Engineering and Technology

Please read this paragraph before you answer the questions.

Engineers use math, science, and creativity to research and solve problems that improve everyone's life and to invent new products. There are many different types of engineering, such as chemical, electrical, computer, mechanical, civil, environmental, and biomedical. Engineers design and improve things like bridges, cars, fabrics, foods, and virtual reality amusement parks. **Technologists** implement the designs that engineers develop; they build, test, and maintain products and processes.

	Strongly Disagree	Disagree	Neither Agree nor Disagree	Agree	Strongly Agree
44. I like to imagine creating new products.	○	○	○	○	○
45. If I learn engineering, then I can improve things that people use every day.	○	○	○	○	○
46. I am good at building and fixing things.	○	○	○	○	○
47. I am interested in what makes machines work.	○	○	○	○	○
48. Designing products or structures will be important for my future work.	○	○	○	○	○

49. I am curious about how electronics work.	○	○	○	○	○
50. I would like to use creativity and innovation in my future work.	○	○	○	○	○
51. Knowing how to use math and science together will allow me to invent useful things.	○	○	○	○	○
52. I believe I can be successful in a career in engineering.	○	○	○	○	○
